# Supplementary material for: Beyond Dunbar circles: a continuous description of social relationships and resource allocation
Source: Sci Rep. 2022 Feb 10;12:2287. doi: 10.1038/s41598-022-06066-1 (PMC8831677; doi:10.1038/s41598-022-06066-1)
Supplement: Supplementary file 1 — Supplementary Information. [file 41598_2022_6066_MOESM1_ESM.pdf]

Beyond Dunbar circles: a continuous description of social relationships and resource allocation. Supplementary Information

Ignacio Tamarit, Angel Sánchez, and José A. Cuesta

1 Mobile phones dataset: comprehensive set of figures

Here we show a comprehensive set of figures complementing the ones presented in section 3.2, where we analysed data from mobile phone calls [1]—see the corresponding section for details and interpretation of the figures. In Fig. S1 we display all the results corresponding to the first time window ($T1$), in Fig. S2 the results corresponding to the second time window ($T2$), in Fig. S3 those corresponding to the third time window ($T3$), and in Fig. S2 the results corresponding to the full 18 months of the study ($T1 \cup T2 \cup T3$).

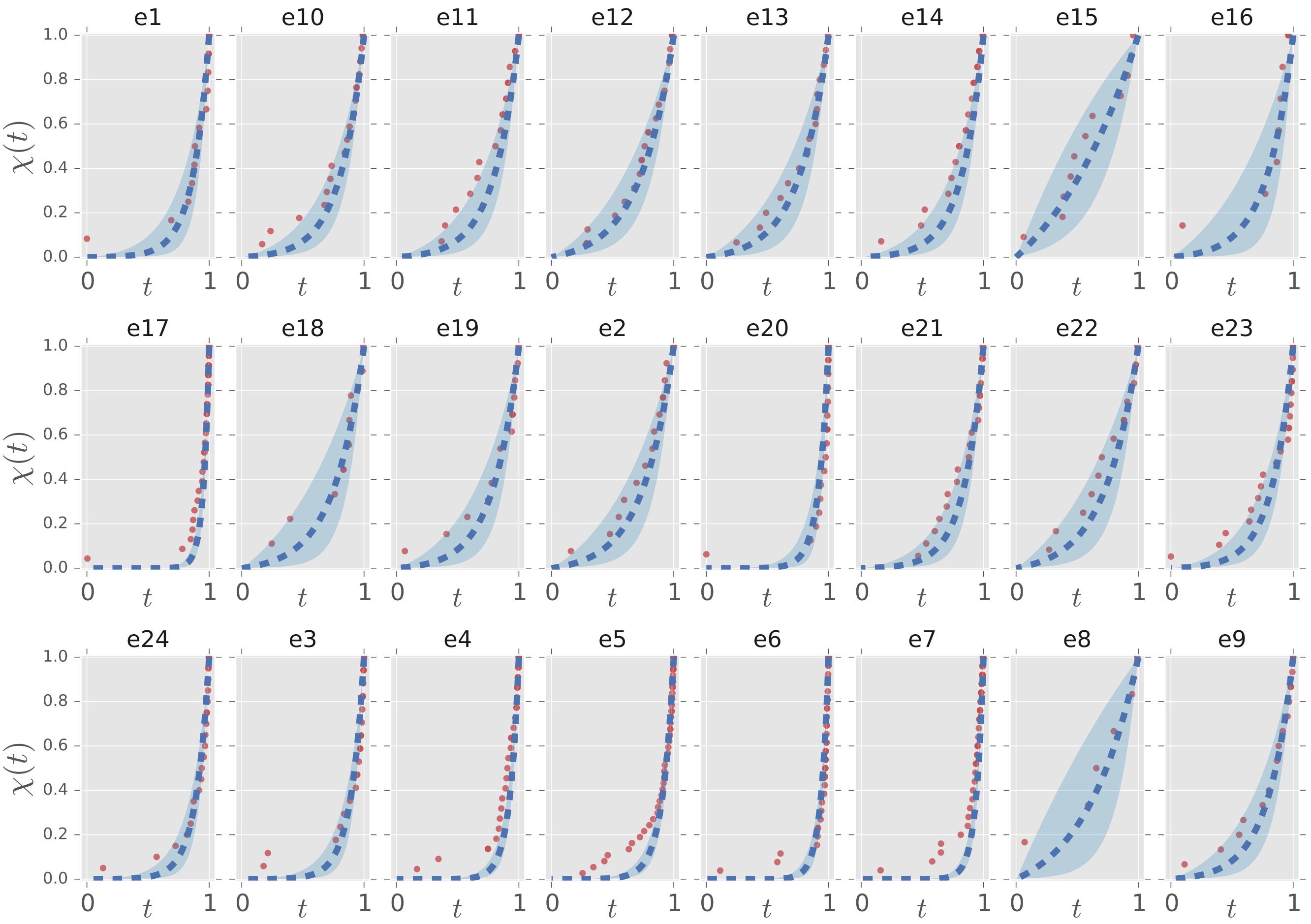

Figure S1: **Complete set of figures for the fittings in $T1$.**

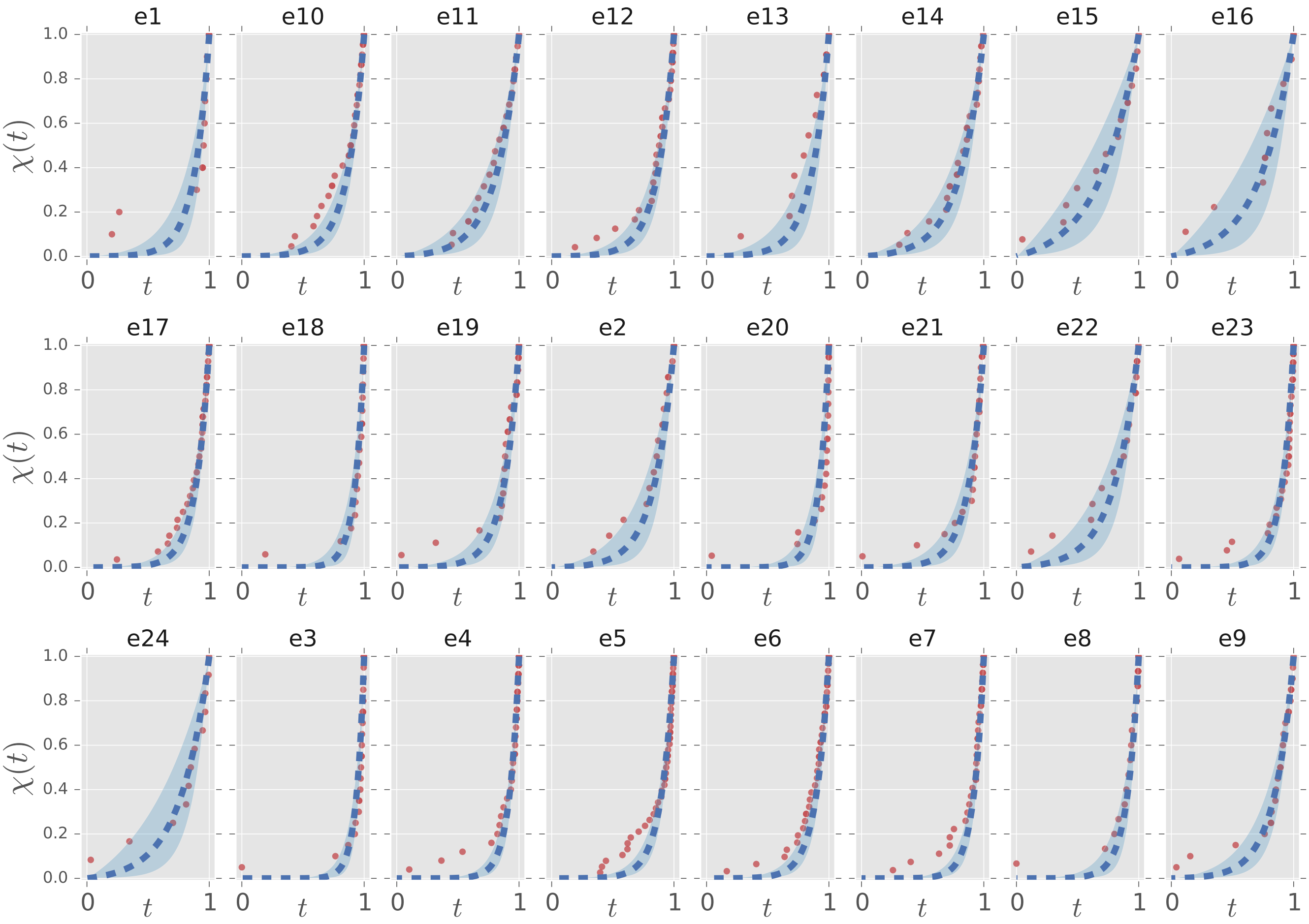

Figure S2: **Complete set of figures for the fittings in $T2$.**

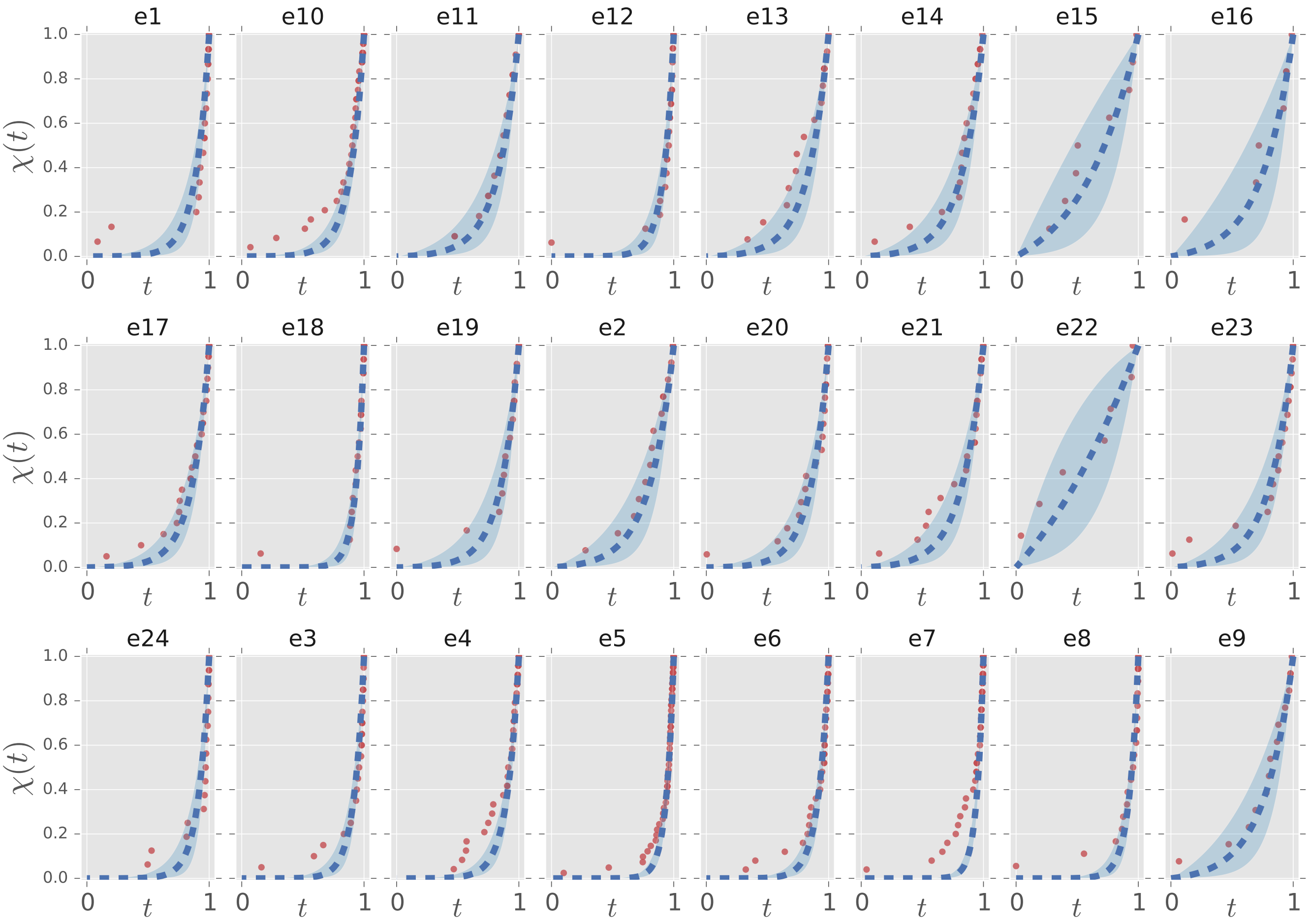

Figure S3: **Complete set of figures for the fittings in $T3$.**

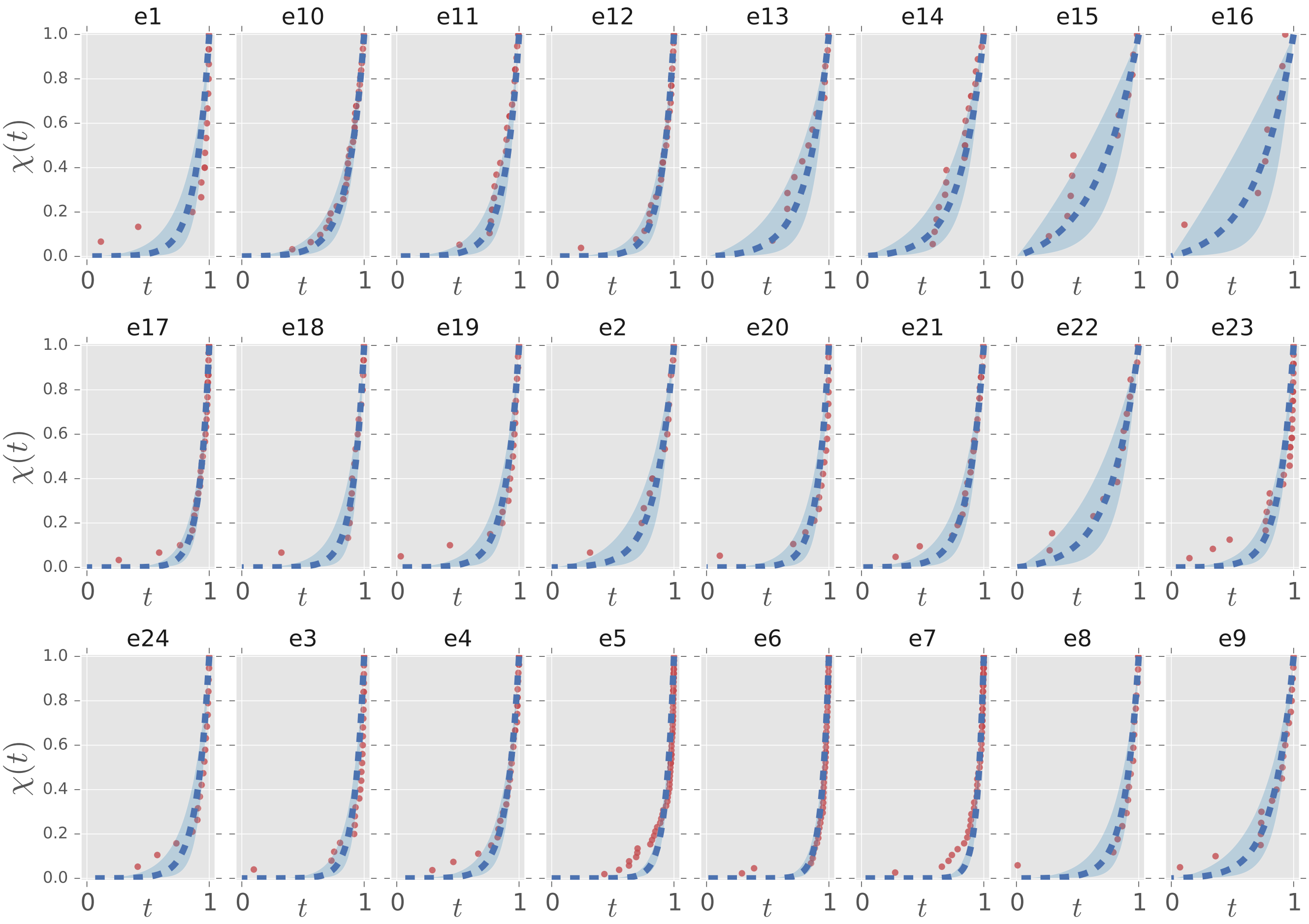

Figure S4: **Complete set of figures for the fittings in $T1 \cup T2 \cup T3$.**

2 Alternative estimation of s_{min} and s_{max}: face-to-face contacts dataset

Here we show an equivalent figure to the one we showed in section 3.3, but considering the sum of the maximum time spent with any alter on each day as s_{max}, and the sum of the minima as s_{min}. The individual fittings are slightly worse (Fig. S5b), and the distribution of the parameter estimates (Fig. S5a) is centred around a higher value ($\eta \approx 14$) than in the figure shown in the main text (Fig. 4a, $\eta \approx 6$). Moreover, filtering out individuals with less than five alters leaves us in this case with a sample of $n = 74$—as opposed to $n = 95$.

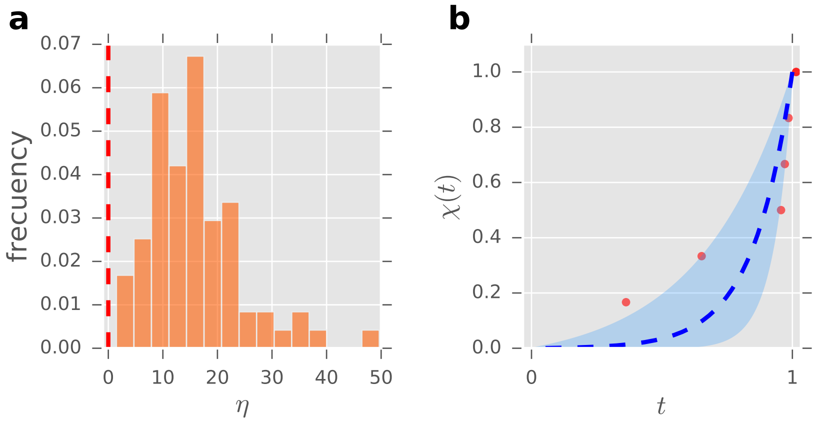

Figure S5: **Summary of the results for the face-to-face contacts dataset. a,** Distribution of the parameter estimates for the face-to-face contacts dataset ($n = 74$). The red, dashed line marks the change of regime $\eta = 0$; $mean = 16.01$, $median = 14.81$, $mode = 14.33$, $std = 8.57$ **b,** Example of fitting for an individual exhibiting the standard regime (chosen at random from those with $3 < \eta < 9$). Solid dots represent experimental data, blue dashed lines represent the graph of equation (9) with the corresponding estimated parameter, and shaded regions show the 95% confidence interval for that estimate (see section 3.1). Estimated $\eta = 8.54$, 95% confidence interval $(4.67, 15.29)$, $\tilde{L} = 11$.

3 Examples of fittings: face-to-face contacts dataset

Here we show 24 examples of fittings for individuals in the face-to-face contacts dataset—sampled at random from the entire population.

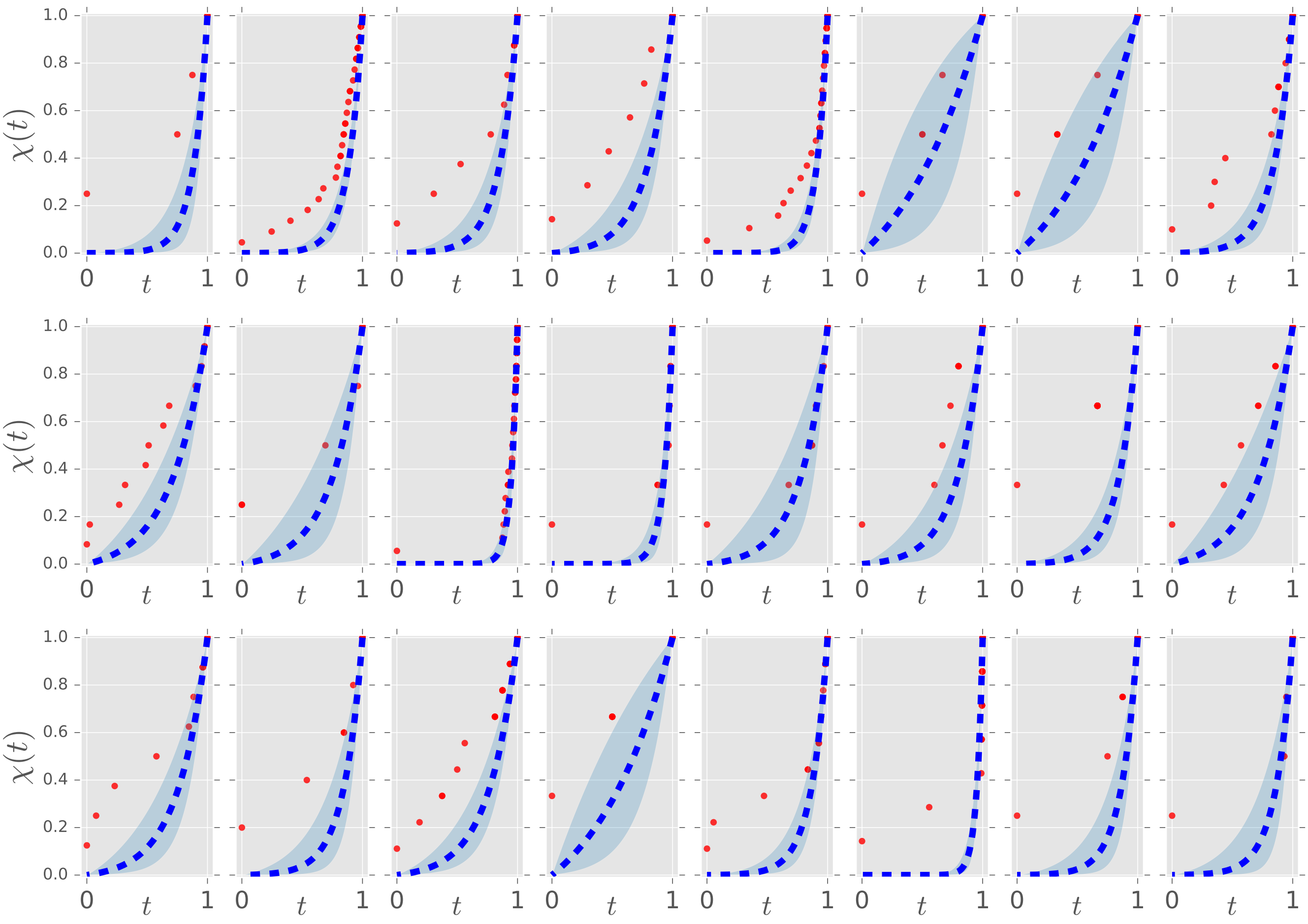

Figure S6: **Examples of fittings for the face-to-face contacts dataset.**

4 Examples of fittings: Facebook dataset

Here we show 24 examples of fittings for individuals in the Facebook dataset—sampled from the entire population.

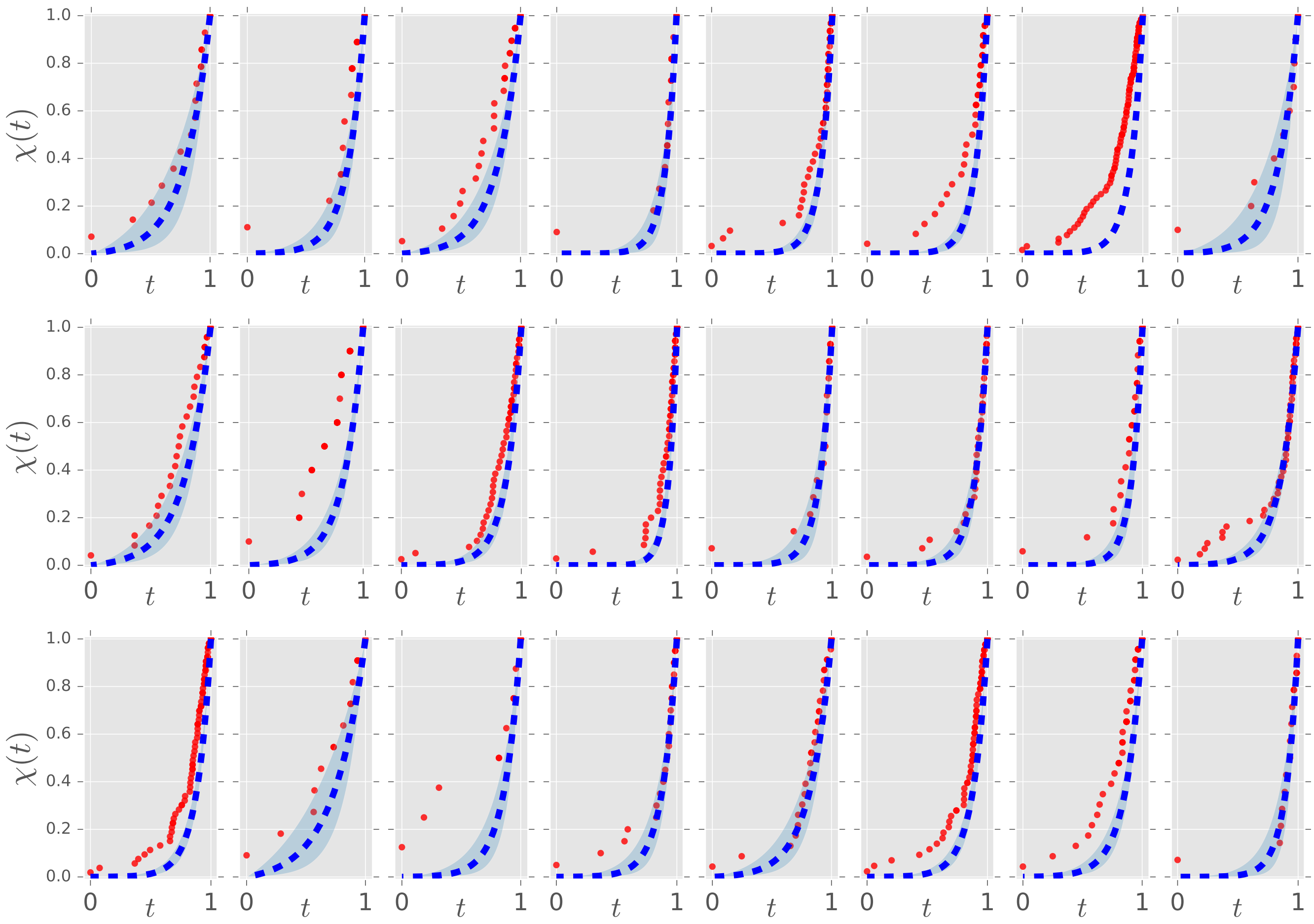

Figure S7: **Examples of fittings for Facebook dataset.**

References

[1] J. Saramäki, E. A. Leicht, E. López, S. G. Roberts, F. Reed-Tsochas, and R. I. Dunbar. Persistence of social signatures in human communication. *Proceedings of the National Academy of Sciences USA*, 111:942–947, 2014.
